# Prognostic value of baseline total metabolic tumour volume of ^18^F-FDG PET/CT imaging in patients with angioimmunoblastic T-cell lymphoma

**DOI:** 10.1186/s13550-021-00807-5

**Published:** 2021-07-15

**Authors:** Huanyu Gong, Tiannv Li, Jianyong Li, Lijun Tang, Chongyang Ding

**Affiliations:** 1 The Department of Nuclear Medicine, The First Affiliated Hospital of Nanjing Medical University, Jiangsu Province Hospital , Nanjing, China; 2 The Department of Haematology, The First Affiliated Hospital of Nanjing Medical University , Jiangsu Province Hospital , Nanjing, China

**Keywords:** Angioimmunoblastic T-cell lymphoma, Prognosis, ^18^F-FDG, Baseline PET/CT

## Abstract

**Purpose:**

The aim of this study was to explore the prognostic value of baseline metabolic parameters of ^18^F-FDG PET/CT imaging in patients with angioimmunoblastic T-cell lymphoma (AITL).

**Materials and methods:**

Fifty-six AITL patients (average age 64.0 ± 1.3 years) diagnosed pathologically from August 2009 to August 2019 were enrolled in this retrospective study. The total metabolic tumour volume (TMTV), total lesion glycolysis (TLG), maximum standardized uptake value (SUVmax), and correlated clinical characteristics were collected and analysed. TMTV was computed with the 41% SUVmax threshold method. The chi-square test or Fisher’s exact probability method was used to compare clinical characteristics. Kaplan–Meier curves were used to describe progression-free survival (PFS) and overall survival (OS). The log-rank test was used to analyse the difference within groups. The statistically significant factors in the univariate regression analysis were incorporated into the Cox risk proportional regression model for multivariate survival analysis.

**Results:**

The TMTV cut-off value was 514.6 cm^3^ from the ROC curve analysis. Forty (71.4%) patients progressed and 31 (55.4%) patients died within a median follow-up time of 19.1 (interquartile range 7.8–34.6) months. The 1-year and 3-year PFS rates were 42.9% and 30.1%, and the 3-year and 5-year OS rates were 45.9% and 34.4%, respectively. Univariate survival analysis showed that high TMTV and TLG may be the factors contributing to poor PFS and OS. Multivariate analysis showed that TMTV and prognostic index for T-cell lymphoma (PIT) were independent parameters for PFS and OS in AITL patients. TMTV, combined with PIT, may have better risk stratification performance than TMTV alone.

**Conclusions:**

Baseline TMTV and PIT were independent prognostic predictors in AITL patients. The combination of TMTV and PIT can facilitate prognostic stratification and contribute to personalized therapy.

## Introduction

Angioimmunoblastic T-cell lymphoma (AITL) is the second most common subtype of peripheral T-cell lymphoma (PTCL) with distinct pathological features and accounting for approximately 15–20% of PTCLs [1]. This malignancy arises from follicular helper T cells and its pathogenesis is not completely understood. Several theories suggest that it may be related to inflammation and immune abnormalities caused by viral infections, such as the Epstein–Barr virus [2, 3]. AITL is clinically characterized by rapid progression and a dismal prognosis. Although chemotherapy, targeted therapy, immunotherapy, and other therapeutic methods have been applied in the treatment of AITL, the 5-year overall survival (OS) rate is about 32–33% [4]. Presently, two scoring systems, the international prognostic index (IPI) and the prognostic index for T-cell lymphoma (PIT), have been proposed in PTCL, but a few studies have shown that the two indexes have limitations in prognostic evaluation and risk stratification of AITL, because of the obvious heterogeneity of PTCL [5–7]. For example, the prognosis of anaplastic lymphoma kinase (ALK)-positive anaplastic large cell lymphoma (ALCL) is significantly better than that of other subtypes of PTCL. Therefore, novel markers and effective prognostic models for AITL still need to be explored and established.

^18^F-FDG PET/CT, which integrates anatomical and functional imaging, has great value in staging, response monitoring, and prognostication assessments of lymphoma, especially Hodgkin’s lymphoma (HL) and FDG-avid non-Hodgkin’s lymphoma (NHL), such as diffuse large B-cell lymphoma (DLBCL) [8–10]. AITL usually manifests as whole-body lymphadenopathies with highly avid FDG uptake in PET/CT imaging [11]. Moreover, several studies have shown that baseline PET/CT metabolic parameters, such as the total metabolic tumour volume (TMTV) and total lesion glycolysis (TLG), have good prognostic value for PTCL. Thus, baseline PET/CT metabolic parameters may have a predictive value for the prognosis of AITL. At present, few studies about AITL have been conducted. Therefore, our study aimed to explore the prognostic value of baseline quantitative metrics derived from PET/CT scans in newly diagnosed AITL patients.

## Materials and methods

### Study cases and clinical data

Patients newly diagnosed with AITL who underwent pretreatment whole-body ^18^F-FDG PET/CT examination between August 2009 and August 2019 were enrolled in this retrospective study. Inclusion criteria are (a) ≥ 18 years old; (b) diagnosed as AITL pathologically according to 2016 WHO criteria [12]; (c) ^18^F-FDG PET/CT performed before any treatment; (d) no glucocorticoid, colony-stimulating factor, or other drugs that could facilitate extramedullary haematopoiesis given 1 week before imaging; (e) positive lesions found on PET imaging; and (f) anthracycline-based chemotherapy accepted, and the chemotherapy programme was CHOP (cyclophosphamide, doxorubicin, vincristine, prednisone) or CHOP plus etoposide (CHOPE). Exclusion criteria are (a) concurrently suffering from other malignant tumours; (b) in the acute phase of infection or uncontrolled chronic inflammation; (c) AITL lesions surgically resected; (d) poor image quality; and (e) incomplete clinical data. Clinical parameters including sex, age, platelet (PLT) count, serum lactic dehydrogenase (LDH), β2-microglobulin (β2-MG), Eastern Cooperative Oncology Group Performance Status (ECOG-PS), bone marrow biopsy (BMB), Ann Arbor staging, and B symptoms were collected from medical records. This study complies with the principles of the Declaration of Helsinki. Approval was obtained from the Ethics Committee of Jiangsu Province Hospital, the First Affiliated Hospital of Nanjing Medical University.

### Imaging protocol

^18^F-FDG was produced automatically by a cyclotron (General Electric, GE, USA) with an automatic synthesis module. All radiochemical purity was more than 95%. All patients fasted for more than 6 h before scans, and fasting blood glucose was kept at less than 7.0 mmol/L. The imaging agent ^18^F-FDG was injected intravenously at 3.70–5.55 MBq/kg, and the examination was performed after the drug was in the body for 60 min in a quiet state. Whole-body PET/CT images were obtained by a Biograph 16-slice High-Resolution PET/CT (Siemens, Germany) scanner in 3-dimensional mode and included a whole-body PET scan (2 min/bed position, 6–7 bed positions in all) and a CT scan (120 kV and 80 mA). The scan range was from the skull to the upper femur, and if necessary, the extremities were scanned. All metabolic images were reconstructed by an iterative method (matrix, 256 × 256; slice thickness, 5 mm) to obtain PET images, and CT acquisition was used for attenuation correction. Finally, PET, CT, and PET/CT fusion images were obtained and saved.

### Image interpretation

All images were interpreted in DICOM format by two experienced nuclear medicine diagnostic physicians blinded to the patient’s clinical outcome. Metabolic evaluation software (the Beth Israel PET/CT viewer plugin for FIJI) [13] was used to semiautomatically delineate the regions of interest (ROIs) in whole-body lesions (including all nodal and extranodal lesions) with a 41% SUVmax threshold method recommended by the European Association of Nuclear Medicine [14], and the ROIs of the brain, kidney, ureter, bladder, and intestine were manually deleted which may be drawn due to physiological or inflammatory uptake. Then, the mean standardized uptake value (SUVmean) and metabolic tumour volume (MTV) of every lesion were obtained. TMTV was calculated by summing the MTVs of all lesions, and the TLG was the sum of all products of each MTV and SUVmean. The pharyngeal lymphatic ring was excluded in the scope of ROI delineation because it was a challenge to accurately determine whether it was infiltrated by lymphoma by using PET imaging alone. In addition, the spleen, pharyngeal lymphatic ring, nasopharynx, and thymus were classified as intranodal areas. The parameter of SUVmax was the highest SUV measured in every lesion site. Bone marrow involvement was included in volume measurement only if it was confirmed by BMB. A vertical diameter of the spleen > 13 cm or a ratio of spleen SUVmax (focal or diffuse uptake) to liver background > 1 was considered spleen involvement [15].

### Surveillance

The follow-up deadline was 1 October 2020. Progression-free survival (PFS) is defined as the duration from the diagnosis of the disease (confirmed by pathological results) to the progression or recurrence of the disease, death due to various reasons, or the end of follow-up. Overall survival (OS) is the duration from the diagnosis of the disease to death due to various reasons or the end of follow-up.

### Risk evaluation methods of IPI and PIT

IPI and PIT scoring systems were calculated to make risk assessments. The IPI scoring system includes five factors: age > 60, ECOG-PS > 1, elevated LDH level, Ann Arbor stage III–IV and extranodal involvement sites > 1. Each factor was worth 1 point. Four risk groups were defined by IPI: low risk (0–1 point), low-intermediate risk (2 points), high-intermediate risk (3 points), and high risk (4–5 points). PIT includes four factors: age > 60, ECOG-PS > 1, bone marrow involvement, and elevated LDH level. Similar to the IPI, four risk groups were defined by PIT: low risk (0 points), low-intermediate risk (1 point), high-intermediate risk (2 points), and high risk (3–4 points).

### Statistical analysis

Statistical analysis was performed by SPSS ver. 26.0 (IBM, USA) software. Continuous variables were represented by the mean ± standard deviation (SD) or median (interquartile range), and categorical variables were represented by count (percentage). With OS as the outcome event, receiver-operating characteristic (ROC) curves were used to obtain the cut-off values for the SUVmax, TMTV, and TLG with OS as the outcome event. The areas under the curve (AUC) value of every ROC curve were calculated, and the median was used as the cut-off value for grouping if AUC < 0.5. Kaplan–Meier curves were used to describe PFS and OS, and the log-rank test was used to analyse the difference. The statistically significant factors in the univariate survival regression analysis were incorporated into the Cox proportional hazards regression model for multivariate survival analysis to obtain the hazard ratio (HR) and 95% confidence interval (CI). Two-sided *p* < 0.05 was considered statistically significant.

## Results

### Clinical characteristics of patients

Fifty-six patients were included in this study and the clinical characteristics of patients are shown in Table 1. The average age of this group was 64.0 ± 1.3 years (range 43–89 years), and the male to female ratio was 1.4:1. Fifty-three (94.6%) patients were in stage III/IV, and only 3(5.4%) patients were in stage II. Thirty-four (60.7%) patients had extranodal involvement: bone marrow (13 cases), skin (9 cases), lungs (4 cases), parotid glands (3 cases), gastrointestinal tract (2 cases), adrenal glands (2 cases), pleura (2 cases), and liver (1 case). In addition, 46(82.1%) cases were diagnosed with spleen involvement.

**Table 1:** Characteristics of the whole population of AITL

Characteristics	No. (%)
Sex/male	33 (58.9)
Age, mean (range, year)	64.0 (43–89)
B symptoms/yes	36 (64.3)
Ann Arbor stage	
II	3 (5.4)
III	25 (44.6)
IV	28 (50.0)
ECOG-PS > 1	13 (23.2)
Elevated LDH	42 (73.3)
Elevated β2-MG	36 (64.3)
Declined PLT count	35 (62.5)
Declined albumin	41 (73.2)
BMB positive	13 (23.2)
Extranodal involvement/yes	34 (60.7)
Extranodal sites > 1/yes	9 (16.1)
IPI	
0–1	10 (17.9)
2–3	27 (48.2)
4–5	19 (33.9)
PIT	
0	10 (17.9)
1	20 (35.7)
2	18 (32.1)
3	5 (8.9)
4	3 (5.4)

ECOG-PS, Eastern Cooperative Oncology Group Performance Status; LDH, lactate dehydrogenase; β2-MG, β2-microglobulin; PLT, platelet; BMB, bone marrow biopsy; IPI, international prognostic index; PIT, prognostic index for T-cell lymphoma.

### ROC curves and cut-off values of TMTV, TLG, and SUVmax

The medians of TMTV, TLG, and SUVmax were 493.6 (interquartile range 281.0–892.5) cm^3^, 2311.2 (interquartile range 1083.3–4322.2), and 17.3 (interquartile range 12.3–21.6), respectively. The AUCs of TMTV and TLG obtained by ROC curve analysis were 0.708 (95% CI 0.574–0.843, *p* = 0.008) and 0.666 (95% CI 0.523–0.809, *p* = 0.034), respectively. The cut-off values of TMTV and TLG were 514.6 cm^3^ (sensitivity 64.5%, specificity: 72.0%, Youden index = 0.365) and 2141.0 (sensitivity 67.7%, specificity 64.0%, Youden index = 0.317), respectively. The median SUVmax (17.3) was applied as the cut-off value for grouping because the AUC of SUVmax was low (AUC = 0.466, 95% CI 0.313–0.618, *p* = 0.662).

### Comparisons of clinical characteristics according to TMTV

Patients were classified into a high TMTV (> 514.6 cm^3^) and a low TMTV (≤ 514.6 cm^3^) group. High TMTV was usually seen in male AITL patients (*p* = 0.026) and was associated with higher ECOG-PS scores (*p* = 0.003), PLT counts (*p* = 0.032), Ann Arbor stage (*p* = 0.001) and PIT scores (*p* = 0.015) (Table 2). TMTV of patients without progression was lower than progressed patients (medians 333.9 cm^3^ vs 607.0 cm^3^, *p* = 0.018), and the difference could be seen in patients lived and died as well (medians 419.9 cm^3^ vs 697.7 cm^3^, *p* = 0.008).

**Table 2:** Comparison of patient clinical characteristics with TMTV

Characteristics	No. of patients (*n* = 56)	TMTV		
		Low (*n* = 29)	High (*n* = 27)	*P *value
Sex, female/male	23/33	16/13	7/20	0.026
Age, ≤ 60/ > 60	20/36	13/16	7/20	0.140
B symptoms, no/yes	20/36	12/17	8/19	0.359
LDH, normal/elevated	14/42	10/19	4/23	0.089
ECOG-PS,0–1/2–5	43/13	27/2	16/11	0.003
PLT count, normal/declined	35/21	22/7	13/14	0.032
Albumin, normal/declined	15/41	9/20	6/21	0.457
β2-MG, normal/elevated	20/36	11/18	9/18	0.720
BMB, negative/positive	43/13	25/4	18/9	0.084
Ann Arbor stage II–III/IV	28/28	21/8	7/20	0.001
Extranodal sites > 1, no/yes	47/9	26/3	21/6	0.227
IPI,0–2/3–5	25/31	13/16	12/15	0.977
PIT,0–1/2–4	36/20	23/6	13/14	0.015

LDH, lactate dehydrogenase; ECOG-PS, Eastern Cooperative Oncology Group Performance Status; PLT, platelet; β2-MG, β2-microglobulin; BMB, bone marrow biopsy; IPI, international prognostic index; PIT, prognostic index for T-cell lymphoma

### Survival analysis of clinical and PET/CT characteristics in AITL

**Fig. 1:**
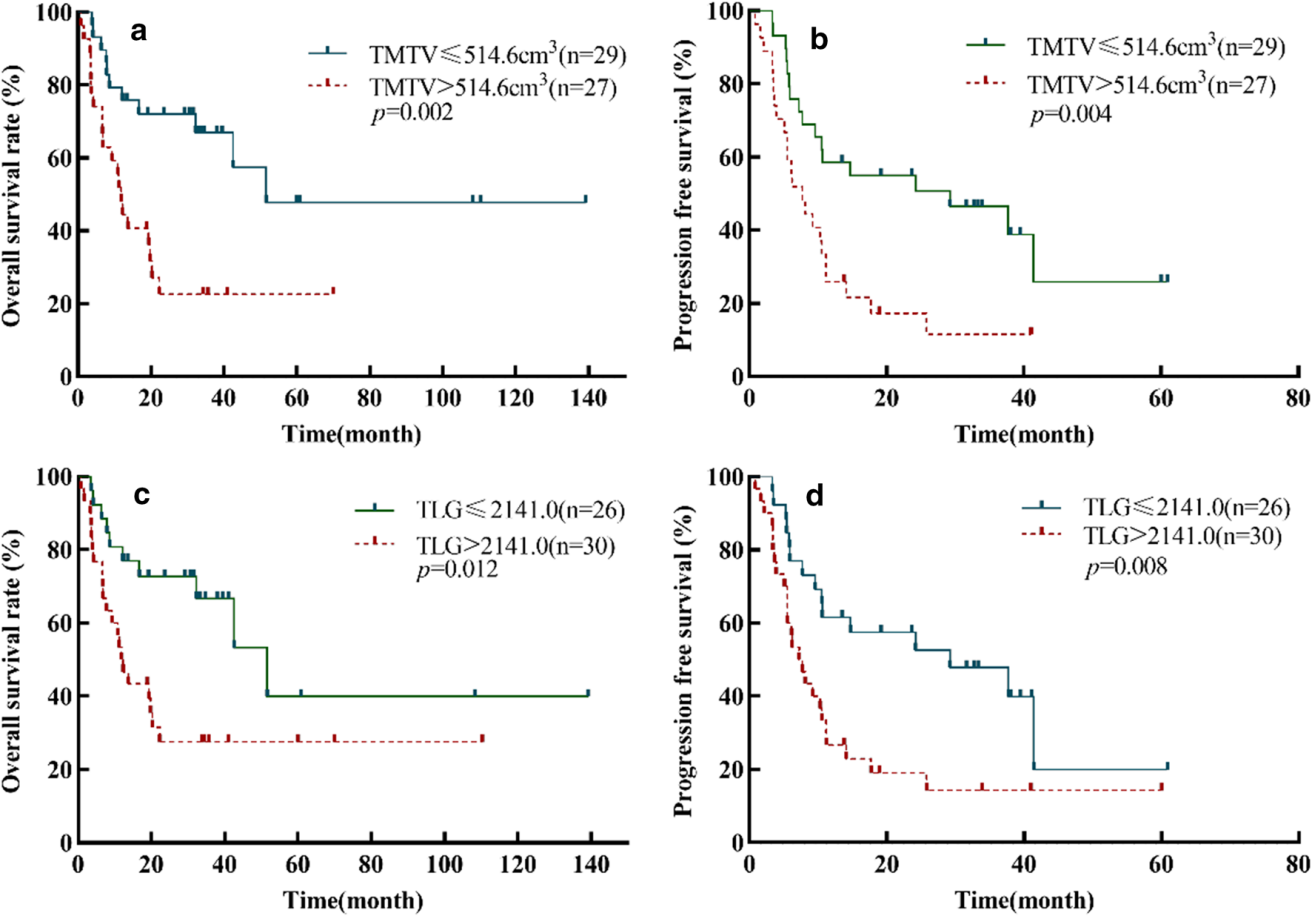
The Kaplan–Meier survival analyses and curves of OS and PFS according to TMTV (**a**, **b**) and TLG (**c**, **d**). *p *value was acquired by the log-rank test

The median follow-up time was 19.1 (interquartile range 7.8–34.6) months. The median PFS and OS times were 10.5 (95% CI 8.4–12.6) months and 22.3 (95% CI 1.5–43.1) months, respectively. Among these patients, 40 (71.4%) progressed and 31 (55.4%) died. The 1-year and 3-year PFS rates were 42.9% and 30.1%; the 3-year and 5-year OS rates were 45.9% and 34.4%, respectively. Kaplan–Meier curves and the log-rank test showed that high TMTV and TLG (> 2141.0) may be risk factors for PFS and OS. Patients with high TMTV and TLG had poor prognosis compared to those with low TMTV and TLG (≤ 2141.0) in PET/CT imaging (Fig. 1).

The results of univariate survival analysis are shown in Table 3. Positive B symptoms, ECOG-PS > 1, low PLT count, BMB positivity, PIT > 1, and high TMTV and TLG were significantly associated with poor PFS and OS. In addition, age > 60 and low albumin level were risk factors for PFS and OS, respectively. Multivariate survival analysis results are shown in Table 4. TMTV was the only independent predictor for PFS and OS, and TLG did not show good prognostic value for AITL. Additionally, PIT and TMTV were introduced into multivariate analysis separately to reduce confounding bias because PIT included four parameters, and results showed (Table 5) that high TMTV and PIT > 1 were independent risk factors for OS and PFS.

**Table 3:** Univariate regression survival analysis for survival and disease progression

Characteristics	1-year PFS rate (%)	HR (95%CI)	*p*	3-year OS rate (%)	HR (95%CI)	*p*
Sex						
Female	56.5			48.1		
Male	33.3	1.445 (0.758–2.756)	0.264	44.5	1.153 (0.559–2.380)	0.699
Age						
≤ 60	65.0			63.5		
> 60	30.6	2.189 (1.080–4.435)	0.030	36.2	2.062 (0.937–4.537)	0.072
B symptoms						
No	60.0			66.9		
Yes	33.3	2.038 (1.007–4.123)	0.048	33.9	2.412 (1.034–5.627)	0.042
Ann Arbor stage						
II/III	50.0			55.4		
IV	35.7	1.401 (0.749–2.620)	0.291	37.5	1.537 (0.751–3.144)	0.239
IPI						
0–2	40.0			50.6		
3–5	45.2	0.712 (0.381–1.331)	0.288	42.6	0.550 (0.270–1.121)	0.100
PIT						
0–1	55.6			61.9		
2–4	20.0	2.543 (1.335–4.845)	0.005	18.0	3.101 (1.521–6.322)	0.002
ECOG-PS						
≤ 1	51.2			55.7		
> 1	15.4	2.915 (1.442–5.890)	0.003	11.5	3.652 (1.714–7.781)	0.001
LDH						
Normal	42.9			64.3		
Elevated	42.9	1.279 (0.586–2.790)	0.536	40.4	1.664 (0.638–4.341)	0.298
β2-MG						
Normal	35.0			49.5		
Elevated	47.2	0.755 (0.394–1.445)	0.396	44.7	0.996 (0.467–2.125)	0.991
PLT						
Normal	19.0			60.7		
Low	57.1	2.585 (1.378–4.849)	0.003	22.2	3.018 (1.480–6.151)	0.002
Albumin						
Normal	46.7			62.0		
Low	41.5	0.917 (0.466–1.806)	0.802	40.2	2.710 (1.034–7.104)	0.043
BMB						
Negative	51.2			52.9		
Positive	15.4	2.384 (1.207–4.708)	0.012	23.1	2.298 (1.092–4.838)	0.028
Extranodal sites > 1						
No	44.7			46.5		
Yes	33.3	1.089 (0.471–2.515)	0.842	44.4	1.380 (0.564–3.375)	0.480
SUVmax						
≤ 17.3	50.0			44.4		
> 17.3	35.7	1.296 (0.695–2.419)	0.415	47.4	0.925 (0.455–1.878)	0.828
TMTV (cm^3^)						
≤ 514.6	58.6			66.9		
> 514.6	25.9	2.481 (1.295–4.754)	0.006	22.6	3.314 (1.476–6.655)	0.003
TLG						
≤ 2141.0	61.5			66.6		
> 2141.0	26.7	2.351 (1.226–4.509)	0.010	27.6	2.543 (1.193–5.421)	0.016

IPI, international prognostic index; PIT, prognostic index for T-cell lymphoma, ECOG-PS, Eastern Cooperative Oncology Group Performance Status; LDH, lactate dehydrogenase; β2-MG, β2-microglobulin, PLT, platelet, BMB, bone marrow biopsy, SUVmax, maximum standard uptake value; TMTV, total metabolic tumour volume, TLG, total lesion glycolysis

**Table 4:** Multivariate analysis of risk factors of overall survival and progression-free survival

	Characteristics	HR (95% CI)	*p*	Characteristics	HR (95% CI)	*p*
OS	TMTV > 514.6 cm^3^	2.586 (1.174–5.696)	0.018	TLG > 2141.0	–	–
	Low albumin	–	–	Low albumin	–	–
	Low PLT	–	–	Low PLT	–	–
	BMB positive	–	–	BMB positive	–	–
	ECOG-PS > 1	2.826 (1.285–6.216)	0.010	ECOG-PS > 1	3.652 (1.714–7.781)	0.001
	B symptoms	–	–	B symptoms	–	–
PFS	TMTV > 514.6 cm^3^	2.333 (1.205–4.516)	0.012	TLG > 2141.0^a^	–	–
	Low PLT	2.425 (1.283–4.584)	0.006	Low PLT	2.053 (1.029–4.095)	0.041
	BMB positive	–	–	BMB positive	–	–
	Age > 60	–	–	Age > 60	–	–
	ECOG-PS > 1	–	–	ECOG-PS > 1	2.107 (0.976–4.547)	0.058
	B symptoms	–	–	B symptoms	–	–

PFS, progression-free survival; OS, overall survival; HR, hazard ratio; CI, confidence interval; ECOG-PS, Eastern Cooperative Oncology Group Performance Status; PLT, platelet; BMB, bone marrow biopsy; TMTV, total metabolic tumour volume; TLG, total lesion glycolysis

^a^TMTV and TLG were separately incorporated into multivariate survival analysis due to the correlation between TMTV and TLG

**Table 5:** Multivariate analysis of TMTV and PIT of overall and progression-free survival

Characteristics	OS		PFS	
	HR (95% CI)	*p*	HR (95% CI)	*p*
TMTV > 514.6 cm^3^	2.552 (1.183–5.506)	0.017	2.090 (1.064–4.107)	0.032
PIT > 1	2.521 (1.208–5.261)	0.014	2.110 (1.082–4.115)	0.028

PIT was individually incorporated into multivariate analysis for reason that PIT includes four indicators which was partly incorporated into the above analysis

PFS, progression-free survival; OS, overall survival; HR, hazard ratio; TMTV, total metabolic tumour volume

### The value of TMTV and PIT in the prognostic stratification of AITL patients

**Fig. 2:**
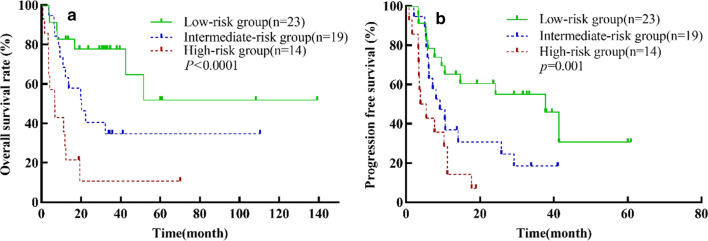
The Kaplan–Meier survival analyses and curves of OS (**a**) and PFS (**b**) according to the combination of TMTV > 514.6 cm^3^ and PIT > 1. *p *value was acquired by the log-rank test

Our analysis showed that both TMTV and PIT can be used as independent prognostic predictors of AITL. Therefore, we combined PIT and TMTV to divide this group of AITL patients into three risk groups: low-risk group (TMTV ≤ 514.6 cm^3^ and PIT ≤ 1), intermediate-risk group (TMTV > 514.6 cm^3^ or PIT > 1), and high-risk group (TMTV > 514.6 cm^3^ and PIT > 1), to explore prognostic stratification efficacy on AITL patients. The results showed 23 cases were low risk, 19 cases intermediate risk, and 14 cases high risk. The 1-year PFS rates for the groups were 65.2%, 36.8%, and 14.3%, and the 3-year OS rates were 77.7%, 34.7%, and 10.7%, respectively. The differences between each group were statistically significant (OS: *χ*^2^ = 18.046, *p* < 0.0001; PFS: *χ*^2^ = 13.853, *p* = 0.001) (Fig. 2). So, the combination of PIT and TMTV can stratify the prognosis of AITL patients better than TMTV alone.

## Discussion

SUVmax was not considered to have significant prognostic value in this study and similar results were also reflected by Xia et al. [16] and Kitadate et al. [17]. The exact prognostic value of pretreatment SUVmax for AITL remains controversial, though the study performed by Zhou et al. showed that pretreatment SUVmax had independent prognostic value in PTCL [18]. Due to the strong heterogeneity of PTCL, the prognosis of different pathological subtypes varies greatly, so its prognostic value for AITL cannot be summarized. Wang et al. studied the prognostic value of baseline SUVmax on AITL in a small sample (23 cases) and pointed out that SUVmax could not be used as an independent risk factor for AITL prognosis (*p* = 0.770) [19]. Our results were similar to Wang’s, and SUVmax was not associated with prognosis by univariate analysis. The possible reasons we considered were that SUVmax represents the FDG uptake of a single voxel in the lesion, while AITL patients often have multiple lesions, and SUVmax cannot represent the overall metabolic level of all lesions. In addition, SUVmax, as a semiquantitative metric, is susceptible to several uncontrollable factors, such as the partial volume effect, injection time, and blood glucose level. Therefore, the use of SUVmax alone to reflect the biological activity of tumours and to evaluate the prognosis still has some limitations. In contrast, the dynamic changes in SUVmax during treatment may have value in evaluating the prognosis of lymphoma, according to a series of recent studies [20, 21].

The acquisition of TMTV and TLG is different from that of SUVmax. TMTV is calculated by setting a threshold to delineate the volumes of interest of all lesions to obtain the total metabolic volumes. Therefore, as volume metrics, both of them can represent the total tumour burden to a certain extent, reflect the severity of the disease, and have important potential in the prognostic stratification of AITL. A number of studies have shown that baseline TMTV and TLG have ideal prognostic value in lymphoma [18, 22–24]. Cottereau et al. and Zhou et al. used 41% SUVmax as the threshold to study the prognostic value of baseline TMTV and TLG in PTCL [18, 25]. A study by Cottereau et al. included 108 patients with PTCL (43 AITL cases). Multivariate analysis showed that only high TMTV (> 230 cm^3^) was an independent predictor of PTCL (PFS, *p* = 0.0013; OS, *p* = 0.021). Moreover, subgroup analysis between patients with PTCL-NOS and AITL on one hand and patients with ALCL on the other hand also showed that high TMTV was an independent parameter of AITL and PTCL-NOS, but no further analysis of AITL was conducted. A study by Zhou et al. enrolled 51 PTCL patients (8 AITL cases), and results showed that high TMTV (> 62.4 cm^3^) and TLG (> 270.7) were independent prognostic factors for PTCL (PFS, both *p* < 0.001; OS, *p* = 0.008, *p* = 0.001), but they did not perform a subgroup analysis and the sample size of AITL was small. We also used the 41% SUVmax threshold method to obtain TMTV and TLG. Multivariate analysis showed that only TMTV > 514.6 cm^3^ was an independent risk factor for PFS and OS in AITL. It is important to note that TMTV cut-off values vary from study to study while TMTV can be used as an independent prognostic predictor. The variation that leads to the need for subgroup analysis for PTCL may be due to the different imaging protocols, sample size heterogeneity, and pathological subtypes.

Additionally, studies on AITL also haves also shown that age, ECOG-PS, B symptoms, PLT count, LDH, β2-microglobulin, albumin, and other clinical characteristics may affect the prognosis of AITL. However, the results were highly controversial and a consistent conclusion could not be reached [6, 26, 27]. Therefore, it is difficult to establish an AITL-specific prognostic model, and the PIT scoring system is still commonly used for risk stratification or prediction. In this study, we incorporated PIT and TMTV into multivariate analysis and found that PIT > 1 could also be used as an independent marker of AITL. Combined with TMTV and PIT, 56 patients with AITL were divided into low, intermediate, and high-risk groups. Kaplan–Meier survival analysis showed that patients in the intermediate/high-risk group had significantly lower rates of PFS and OS than those in the low-risk group. This indicated that the combination of TMTV and PIT also had satisfactory performance and added value to prognostic stratification in AITL, which was partly confirmed by the studies of Cottereau et al. and Jiang et al. [24, 25].

There are several limitations to this study. First, this study was limited by its retrospective nature. Second, the small sample size might lead to a risk of overfitting. Third, the time span for those patients enrolled in this study was long, and inconsistencies in first-line chemotherapy might have caused bias.

In conclusion, AITL has a low incidence and dismal prognosis, and no specific prognostic model has been established yet. In this study, we retrospectively analysed ^18^F-FDG PET/CT metabolic parameters in patients with AITL before treatment and found that TMTV could be an independent prognostic factor for predicting AITL survival. The combination of TMTV and PIT can help in the development of risk stratification and tailored therapeutic plans.

## Data Availability

The datasets generated and analysed during the current study are available in the Jiangsu Province Hospital, the First Affiliated Hospital of Nanjing Medical University.
